# Using Plant Functional Traits and Phylogenies to Understand Patterns of Plant Community Assembly in a Seasonal Tropical Forest in Lao PDR

**DOI:** 10.1371/journal.pone.0130151

**Published:** 2015-06-26

**Authors:** Manichanh Satdichanh, Jérôme Millet, Andreas Heinimann, Khamseng Nanthavong, Rhett D. Harrison

**Affiliations:** 1 Xishuangbanna Tropical Botanical Garden, Chinese Academy of Sciences, Menglun, Mengla, Yunnan, P.R. China; 2 University of the Chinese Academy of Sciences, Beijing, P.R. China; 3 Faculty of Forestry Science, National University of Laos, Dongdok Campus, Vientiane, Lao PDR; 4 Fédération des Conservatoires botaniques nationaux, Montreuil Cedex, France; 5 Centre for Development and Environment and Institute of Geography, University of Bern, Bern, Switzerland; 6 World Agroforestry Centre, East & Central Asia Regional Office, Kunming, Yunnan, China; 7 Key Laboratory of for Plant Diversity and Biogeography of East Asia, Kunming Institute of Botany, Chinese Academy of Sciences, Kunming, Yunnan, China; Chinese Academy of Forestry, CHINA

## Abstract

Plant functional traits reflect different evolutionary responses to environmental variation, and among extant species determine the outcomes of interactions between plants and their environment, including other plant species. Thus, combining phylogenetic and trait-based information can be a powerful approach for understanding community assembly processes across a range of spatial scales. We used this approach to investigate tree community composition at Phou Khao Khouay National Park (18°14’-18°32’N; 102°38’- 102°59’E), Laos, where several distinct forest types occur in close proximity. The aim of our study was to examine patterns of plant community assembly across the strong environmental gradients evident at our site. We hypothesized that differences in tree community composition were being driven by an underlying gradient in soil conditions. Thus, we predicted that environmental filtering would predominate at the site and that the filtering would be strongest on sandier soil with low pH, as these are the conditions least favorable to plant growth. We surveyed eleven 0.25 ha (50x50 m) plots for all trees above 10 cm dbh (1221 individual trees, including 47 families, 70 genera and 123 species) and sampled soils in each plot. For each species in the community, we measured 11 commonly studied plant functional traits covering both the leaf and wood economic spectrum traits and we reconstructed a phylogenetic tree for 115 of the species in the community using rbcL and matK sequences downloaded from Genebank (other species were not available). Finally we compared the distribution of trait values and species at two scales (among plots and 10x10m subplots) to examine trait and phylogenetic community structures. Although there was strong evidence that an underlying soil gradient was determining patterns of species composition at the site, our results did not support the hypothesis that the environmental filtering dominated community assembly processes. For the measured plant functional traits there was no consistent pattern of trait dispersion across the site, either when traits were considered individually or when combined in a multivariate analysis. However, there was a significant correlation between the degree of phylogenetic dispersion and the first principle component axis (PCA1) for the soil parameters. Moreover, the more phylogenetically clustered plots were on sandier soils with lower pH. Hence, we suggest that the community assembly processes across our site may reflect the influence of more conserved traits that we did not measure. Nevertheless, our results are equivocal and other interpretations are possible. Our study illustrates some difficulties in combining trait and phylogenetic approaches that may result from the complexities of integrating spatial and evolutionary processes that vary at different scales.

## Introduction

Plant functional traits (PFT) represent an evolutionary response of plants to climate, life history, defense, water relations, carbon gain and competition [1,2]. Therefore, PFT are important indicators of ecological strategy and are strongly predictive of ecosystem responses to environmental change. PFT also themselves have strong impacts on ecosystem processes [3]. As a consequence, PFT are central to determining the interactions between plant species and their environment. PFT can be used to identify the degree of ecological divergence among species in an assemblage, which is important not only for understanding the distribution and coexistence of related species, but also for understanding the species diversity-functional diversity relationship [4]. For example, Cornwell et al. [5] showed that PFT played a critical role in determining species abundance among co-occurring species. Specifically, they found a strong relationship between plant species abundance and maximum height and specific leaf area (SLA). Species with low SLA and large maximum height were more abundant compared to co-occurring species with smaller maximum height and higher SLA. Both SLA and maximum height are associated with the successional status of species.

The differences among species that co-occur in an ecological community are the result of modifications to a common ancestor that all species ultimately share. Hence, knowledge of the evolutionary relationships among species is essential to understanding the relationships among organisms with respect to community ecology, community organization, and species interactions. For example, closely related species are generally expected to have strongly overlapping niches and hence to have strong competitive interactions, which should limit coexistence and drive the evolution of divergent traits. Thus the pattern of evolution of a particular trait, whether conserved, divergent or convergent, combined with information on community composition at the local scale, can shed light on the species assembly process [6,7]. For example, finding an association between abundance and relatedness might indicate that phylogenetically conserved characters are influencing local abundance. On the other hand, differing evolutionary histories in different areas has led to occupancy of similar niches by distantly related species (trait convergence) and, because clades many vary in their potential for diversification, these areas will support different numbers of extant species [7].

Webb et al. [7] and Cavender-Bares et al. [8] have demonstrated how the combination of phylogenetic approaches and trait similarities may be used to explain community assembly patterns (Table 1). Trait distributions within local assemblages are expected to depend on the ecological processes responsible for community assembly. Thus, trait clustering is expected to occur when community assembly is predominantly driven by environmental filtering, while trait over-dispersion is expected to occur when community assembly is driven by competitive interactions. With respect to the community phylogenetic structure, environmental filtering should lead to a phylogenetically clustered pattern when traits are conserved, or phylogenetic over-dispersion if they are convergent. Meanwhile, competitive exclusion should lead to phylogenetic over-dispersion when traits are conserved or a random pattern if they are convergent. Hence, by combining information on PFT and phylogenies we can examine community dispersion patterns and test for different outcomes of the community assembly processes at different spatial or evolutionary scales.

**Table 1:** Expected trait and phylogenetic dispersion patterns within a local community according to the dominant ecological assembly process and pattern of trait evolution.

	Trait similarity	Traits phylogenetically conserved	Traits phylogenetically convergent
Environmental filtering	(a) Traits clustering	(c) Phylogenetic clustering	(e) Phylogenetic over-dispersion
Competitive exclusion	(b) Traits over-dispersion	(d) Phylogenetic over-dispersion	(f) Phylogenetic random

(a) If the community assembly is driven by environmental filtering, coexisting species should share similar niches leading to trait clustering. (b) If community assembly is driven by competitive exclusion coexisting species will be phenotypically less similar than expected by chance (trait over-dispersion). (c) When environmental filtering acts on conserved traits, the community phylogenetic pattern is expected to be clustered, whereas (d) competitive exclusion will result in phylogenetic over-dispersion. Conversely, when traits are convergent (e) environmental filtering will lead to a pattern of phylogenetic over-dispersion while (f) competitive exclusion will lead to a phylogenetically random pattern [7,8].

Our study site in central Laos is characterized by complex topography and strong differences in soil conditions over short distances. Three distinct forest types are present at the site, including mixed deciduous forest and dry evergreen Dipterocarp forest at lower elevations, and pine forests at higher elevation. It has been suggest that differences in soils may be responsible for driving the marked turnover in the tree community across the site [9]. Focusing on the two lowland forest types, the aim of our study was to examine this hypothesis, using PFT and phylogenies to investigate community assembly processes. Specifically, we addressed the following questions. (1) Does environmental filtering dominate the community assembly process at our site? (2) Do soil gradients predict trait dispersion patterns across the site? (3) What is the phylogenetic dispersion pattern among local communities?

## Methods

### Study area

Laos is a land-locked country located at the heart of the Indo-Chinese peninsula. Phou Khao Khouay National Park (PKK) is one of eighteen sites in Laos that were designated as protected areas in 1993. PKK (18°14’-18°32’N; 102°38’- 102°59’E) is a mountain range about 40 km northeast of Vientiane at its closest point. It encompasses an area of around 2,000 km². Ang Nam Ngum reservoir, the largest artificial lake in SE Asia, is situated to the Northwest of the PKK mountain range and delimits the park’s boundary on that side. The forest types found within PKK correspond to the mixed deciduous forest (dominated by Fabaceae), dry evergreen Dipterocarp forest, and monodominant coniferous forest (mainly Pinaceae) at higher elevation [9]. Elevation varies from ≤100 m to nearly 1,700m. Average rainfall in the rainy season (May-October) is 3369 mm, while on average only 265 mm of rainfalls from November-April.

### Sampling and floristic data

Data were collected from 11 permanent forest plots located on the eastern side of PKK. These plots covered the mixed deciduous forest and dry evergreen Dipterocarp forest only. Elevation of the plots ranged from 300–450 m. These permanent forest plots were established by the Institude Recherche pour le Developpement (IRD)-France and Faculty of Forestry (FOF) National University of Lao (NUoL) in 2009 [9]. Each plot was 0.25 ha (50 m × 50 m) and was divided into 25 subplots of 10 x 10 m. After stratifying by forest type, the location of plots was randomly assigned. Thus, conditions within plots were relatively homogeneous, compared to the differences among plots. All individual trees with a diameter-at-breast-height (dbh) ≥10 cm were enumerated. Tree dbh, height, and position were recorded and the species identified. Herbarium specimens were collected and deposited at the National Herbarium of Laos, NUoL Faculty of Forestry herbarium and NUoL Faculty of Science herbarium. From the 11 permanent plots a total of 1221 individual trees, including 47 families, 70 genera and 123 species were enumerated.

### Plant functional trait measurement

#### Trait selection

Plant functional trait measurements were recorded for 10 individual trees per species, unless only a smaller number of individuals were available. Only species with a minimum of two individuals were included. For all species at least one individual tree of each species was collected from each plot it occurred in to provide the reliable estimate of mean trait values [3]. Eleven vegetative traits were measured; specific leaf area (SLA), leaf area (LA), leaf thickness (LT), leaf vein density (LVD), wood density (WD), plant height (H), diameter at breast height (DBH), basal area (BA), crown width (CW), leaf nitrogen (N), leaf phosphorous (P) and leaf carbon content (C). Completely developed leaves from sun-exposed branches were collected for measurement of leaf traits [3]. For rarer understory species it was sometimes difficult to locate sun-exposed branches. In such cases only leaves from the least shady branches were sampled and overall less than 15% of leaf samples were taken from shaded individuals. Sixty leaves per individual tree were used for leaf trait measurements. We considered a leaflet as the laminar unit for compounds leaves.

Two key spectra of functional traits, the “leaf economic spectrum” and the “wood economic spectrum”, have been shown to provide striking patterns of correlations among traits and their environment [1,2,10,11]. The traits we used in this study cover both these spectra. SLA is positively correlated with relative growth rate or mass-based maximum photosynthetic rate and is associate with leaf defense and leaf life span [2,3,12]. LA is important for the leaf energy and leaf water balance [2,13]. LT is related to strategies of resources acquisition and use, especially light, water and nutrients [14,15]. LVD is related to leaf temperature control and hydraulics [16]. Plant height is associated with plant life-history strategy, climate and soil resources [3]. Plant height also has strong allometric relationships with DBH, WD, and CW [3,17]. Ackerly & Cornwell [10] found that SLA, LA, WD and tree maximum height co-varied reflecting species distributions across a soil moisture availability gradient. Leaf chemistry (N; P; C) is correlated to the leaf economic spectrum, especially the investment in defense versus resource acquisition [3].

#### Trait measurements

For LA and SLA measurements, all leaves were scanned and ImageJ software was used to calculate leaf area. Next leaves were dried in an oven at 80°C for 48 hrs, and immediately weighed using an electronic balance to a precision of 0.001g. SLA (cm^2^ g^-1^) is defined by fresh leaf area / dry mass.

LT (mm) was measured as leaf base thickness using a digital caliper to a precision of 0.001 mm.

LVD (cm/cm^2^) was calculated as the vein length per unit area [18]. To remove chlorophyll, leaves were soaked in 5% NaOH aqueous solution for 24–40 hrs, then transferred to a 50% EtOH solution for 5–30 min. Next each leaf was rinsed in H_2_O and transferred to ethanol at 30%, 50%, 70%, and 100%, with no more than 5 min at each concentration. After the washing process, a two-step staining procedure was used [19]. Each leaf was stained in 1% safranin aqueous solution for 10–20 min and then a 1% fast green aqueous solution for 2–5 S, before rinsing in H_2_O. Three sections of leaf lamina from the top, middle and bottom were photographed using a Olympus sz61 microscope, and vein length and area were calculated using ImageJ software.

For WD, wood samples were collected using an increment borer and the water displacement method was used to determined fresh volume. The wood samples were then dried in an oven at 105°C for 72 hrs [20] and immediately weighed using an electronic balance to a precision of 0.001g. WD (g/cm^3^) was calculated as dry mass (g) / fresh volume (cm^3^).

CW was measured using a tape-measure across the longest and shortest, diameters of crown. For individual trees with complex or irregular crowns several measurements were taken. CW (m) was estimated as the mean crown diameter.

Tree maximum height was determined using a Suunto clinometer and the distance to the tree was fixed at 30 m.

We regressed the values of H and CW against DBH to control for the effect of overall plant size.

For leaf N, P and C contents were analyzed at Biogeochemical Laboratory, Xishuangbanna Tropical Botanical Garden, Chinese Academy of Sciences using a Carbon and Nitrogen analyzer: Vario MAX CN: ICP-AES/iCAP6300.

### Construction of community phylogeny

Only angiosperm species occurred in our plots. We constructed a phylogeny for these tree species in our community using MEGA5 [21], following the protocol of Hall [22]. Phylogenetic analysis included two genes rbcL and matK. DNA sequences were downloaded from GenBank at the genus level, and then aligned using ClusterW. We then selected a best-fit maximum likelihood model of nucleotide substitution using the model selection function in MEGA5. The model with the lowest Bayesian Information Criterion (BIC) score is considered to describe the best substitution pattern. The maximum likelihood values and branch lengths are presented in the model. Evolutionary history was inferred using maximum likelihood based on the Tamura 3-parameter model [23] and the confidence of nodes examined by bootstrapping and inferred from 500 replicates. The initial trees for the heuristic search were obtained by applying the Neighbor-Join and BioNJ algorithms to a matrix of pairwise phylogenetic distances (PPDs). PPDs were estimated using the Maximum Composite Likelihood approach and then selecting the topology with superior log likelihood value. A discrete Gamma distribution was used to model evolutionary rate differences among sites (5 categories (+G, parameter = 5.6608)). The codon position included 1^st^ + 2^nd^ + 3^rd^ + Noncoding; all positions with less than 95% site coverage were eliminated and <5% alignment gaps were allowed at any position. The final phylogenetic tree was constrained at the ordinal level by the Angiosperm Phylogeny Group classification III [24].

### Phylogenetic signal

The phylogenetic signal was assessed by calculating Blomberg’s K statistic. The K statistic enables one to compare the amount of phylogenetic signal across traits and over a phylogenetic tree [25]. A K value of 1 indicates that trait distribution on the phylogenetic tree completely agrees with a Brownian model of evolution across the phylogeny. K < 1 indicates a convergent trait, while K > 1 indicates a conserved trait. The K statistic test was implemented using the *picante* package in R.

### Soil gradients

Soil sample collection and analyses were conducted by the Sud Expert Plantes Initiative Project [9]. In each plot, one soil sample was collected at two depths, between 0–10 cm and >10 cm depth, from two locations across the diagonal of each plot using a soil auger. Soils were analyzed at the soil analysis laboratory of the National Agriculture and Forestry Research Institute (NAFRI) in Vientiane, Lao PDR. The soil samples were analyzed for pH, organic matter (OM), nitrogen and phosphorus, potassium oxide (K_2_O), particle size and texture.

We used results from the surface soil samples only and used plot mean values to characterize the environmental conditions at the plot level. To reduce the number of variables, we conducted a principle component analysis. Soil parameters were first scaled to a mean of zero and standard deviation of one. PCA1 accounted for 59% of the variance in the measured soil parameters. Sand content was strongly positively correlated with PCA1, while P, K_2_O and soil texture were negatively associated with this axis. PCA2 accounted for a further 25% of the variance in measured soil parameters. pH, K_2_O, clay and silt content were positively correlated with PCA2, while OM and N were strongly negatively correlated with this axis (Supplementary materials, S1 Fig)

### Plant distributions across soil gradients

To examine how the plots were distributed with respect to soil parameters. We conducted Canonical Correspondence Analysis (CCA) of plant community abundance against soil parameters. The CCA analysis and axes significance tests were implemented in the *vegan* package in R.

### Phylogenetic and trait community structure

Both phylogenetic community structure analyses and trait dispersion analyses were implemented in R v3.0.1 [26] using *picante*, *APE*, *Geiger*, *ade4*, *spacodiR*, *FD* and *cluster* packages. To investigate variation in community structure with spatial scale, we repeated all analyses at the plot (0.25 ha) and subplot (0.01 ha) scales.

Mean Phylogenetic Distance (MPD) and Net Relatedness Index (NRI) were calculated [7]. MPD is based on the mean pairwise phylogenetic distance between all species in each community, weighted by either species abundance or basal area. Basal area weighted metrics often reflect the relative importance of biotic and abiotic interactions better than stem density derived metrics [27–30]. The NRI is then calculated by comparing the observed phylogenetic relatedness values (MPD) with a null community. The null community was generated by random allocations of individuals to plots (n = 999) for all species sampled in the studied community with abundance >0 (null model 1s), as recommended by Hardy [27]. Thus, the species abundance distribution overall community is held constant. NRI values were calculated as follows: NRI = -1((MPD_observed_−MPD_random_) / MPD_observed_). Thus, positive NRI values indicate phylogenetic clustering while negative NRI values indicate phylogenetic over-dispersion. As the values are centered to zero and standardised by the standard deviation, absolute values >1.96 are significantly phylogenetically structured at P<0.05 [28].

For trait analyses, we first examined the pairwise correlations of all traits across all species and selected uncorrelated traits (Pearson's r < 0.50) for further analyses. For trait dispersion analyses, we log transformed (log10) trait values to improve the normality of data before constructing a data matrix and calculating the Euclidean distance across all species. We then ran cluster analysis on this matrix using the hclust algorithm in R with the default options. Next we calculated the functional dispersion (FDis) from the trait dendrogram, as recommended by Aiba et al. [29]. FDis is the mean distance of individual species to the centroid of all species in the community, weighted by species relative abundance or basal area [30]. FDis values were calculated as *FDis* = *(Σa*_*j*_ *z*_*j*_) */ Σa*_*j*_

Where *a*_*j*_ is the abundance of species *j* and *z*_*j*_ is the distance of species *j* to the weighted centroid. The abundance weighted or basal area weighted analyses are thought to yield more consistent trait dispersion patterns than analyses based on presence-absence [31]. FDis values were calculated by using the *fdisp* function of the *FD* package in R. To test whether FDis of co-occurring species within local communities were different from the null expectation, we used the same null communities as used in the analysis of phylogenetic community structure. We calculated standardized effect sizes of FDis (ZFDis) [32, 33] for both individual and multivariate traits. For multivariate traits analysis we combined the selected traits (Table 2) across all species in the communities and then calculated FDis.

**Table 2:** Phylogenetic signal of plant functional trails for trees at PKK.

Traits	K	p-value
WD	0.04	0.26
LA	0.049	0.01
SLA	0.043	0.08
LT	0.035	0.5
LVD	0.051	0.01
DBH	0.034	0.65
H	0.054	0.003
CW	0.045	0.094
C	0.043	0.54
N	0.039	0.66
P	0.046	0.31

K = Bloomberg’s K and the p-value indicates the probability of obtaining that K value or a more extreme one. All traits had K values of < 1 indicating evolutionary convergence. However, this was only significant for H, LA and LVD (P < 0.05).

LA = leaf area; SLA = specific leaf area; LT = leaf thickness; LVD = leaf vein density; DBH = diameter-at-breast-height; H = height; CW = crown width; BA = basal area; C = leaf carbon; N = leaf nitrogen; P = leaf phosphorus.

ZFDis were calculated as follows: ZFDis = (FDis_obs_−Fdos_null_) / sdFDis_null_. Where FDis_null_ is the mean value of FDis from the randomly assembled communities and sdFDis_null_ is the standard deviation. Similar to calculation of NRI values, we weighted ZFDis by relative abundance and basal area across all species in the given communities. Positive ZFDis values indicate trait dispersion is smaller than expected by chance (clustering) and negative ZFDis values indicate trait dispersion is larger than expected by chance (over-dispersion) [33–35]. Similarly to NRI, the ZFDis values will be significantly different from zero when the absolute value is >1.96.

To test how phylogenetic structure and trait dispersion patterns varied across soil gradients, we examined how well the soil PCA scores (PCA1 and PCA2) predicted NRI and ZFDis values using least squares regression. ZFDis values were log transformed (log10) before analysis. We used the standard model plotting functions in R to examine model fit.

### Functional and phylogenetic similarity analysis

To examine the functional trait similarity, we first ran a hierarchical agglomerative clustering analysis based on the Sorenson similarity index and used the complete linkage method for group identification. Similar to the phylogenetic structure analysis, the null community was generated by random allocations of individuals to plots (n = 999) [27] and included all species sampled in the studied community with abundance >0 (null model 1s). The index of trait similarity among communities was calculated by using the *phylosor* function in the *picante* package in R. To examine phylogenetic similarity between communities, we followed the same procedure as above, but substituted a phylogenetic tree for the trait cluster dendrogram.

To examine the relationship between functional trait or phylogenetic similarity and environmental similarity. We calculated a distance matrix of the soil parameters between communities based on the Euclidean distance. Then we used Mantel tests to examine the relationship between the functional trait or phylogenetic distance matrices and the soil parameter distance matrix. These analyses were implemented in *picante* package in R.

### Data availability

All data, including secondary datasets for phylogenetic and trait dispersion analyses, and the r-scripts used for analysis have been archived and can be freely downloaded from www.datadryad.org (doi:10.5061/dryad.6v0gd).

### Ethics statement

Our study did not involve any research on human subjects or require access to land or other resources owned by indigenous peoples. Permission to work in PKK was obtain from the PKK National Park Authority through Sud Expert Plantes Initiative project coordinated by the Institut de Recherche pour le Développement France and the Faculty of Forestry of the National University of Laos. The research did not require the uprooting of plants; hence no special permits were required for the collection of plant material. The research did not require the removal of collected plant materials from Laos and vouchers of all species were deposited at the National Herbarium of Laos, Faculty of Forestry herbarium and Faculty of Science herbarium, NUoL.

## Results

### Plant distributions with respect to soil parameters

**Fig 1:**
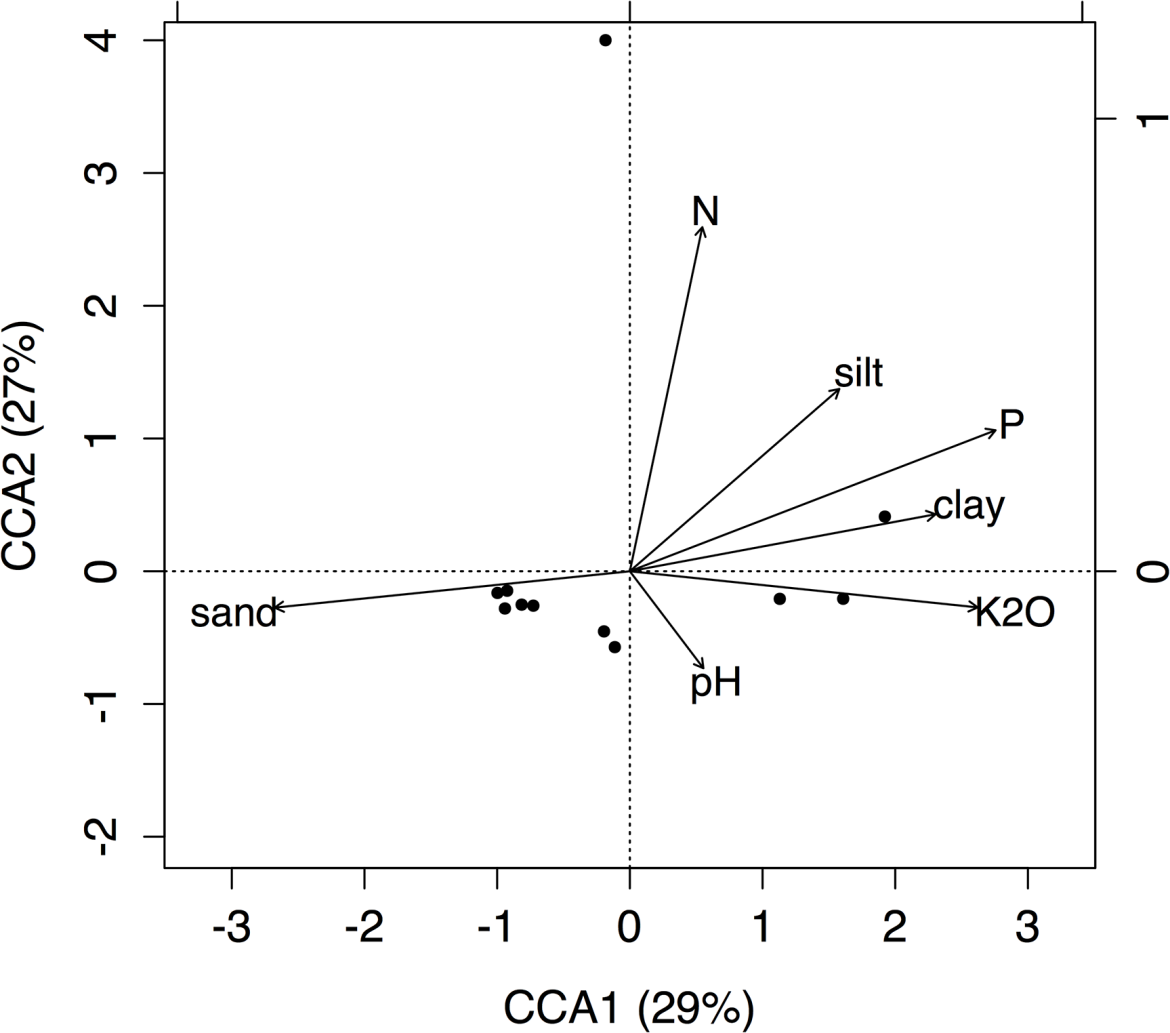
Canonical Correspondence Analysis (CCA) of plant communities with five selected soil parameters in PKK. CCA1 explained 29% of the total inertia and CCA2 explained a further 27% of the total inertia. However, axis significance tests indicated that only CCA1 represented a significant association between tree community composition and soil parameters (CCA1 p = 0.04, CCA2 p = 0.11).

CCA axis 1 (CCA1) explained 29% of the total inertia in angiosperm species composition; K_2_O, clay, silt, P, pH and N were positively associated with CCA1, while sand was negatively associated with this axis. CCA axis 2 (CCA2) explained a further 27% of the total inertia in species composition; clay, silt, P and N were positively associated with CCA2, while pH, sand and K_2_O were negatively associated with this axis. However, the axis significance test found that only CCA1 evidenced a significant association between tree community composition and soil parameters (CCA1 p = 0.04, CCA2 p = 0.11) (Fig 1).

### Community phylogeny

The phylogenetic tree contained 115 species out of 123 angiosperm species in the tree community at PKK. Data on the remaining 8 species were not available from GeneBank. These species comprised only 10.2% of the individuals and were excluded from phylogenetic analyses. The phylogenetic tree and list of species used in the phylogenetic reconstruction with their accession number in GenBank are given in the supplementary material (S1 Table and S2 Fig).

### Plant functional traits

SLA was weakly correlated with LT (Pearson's r = 0.39, P < 0.001, S2 Table). However, neither parameter was correlated with any other (S2 Table). As expected for allometrically linked traits, tree DBH, H and CW were all strongly correlated (all Pearson's r > 0.63, S2 Table). Also, as predicted from the leaf economic spectrum, leaf carbon, nitrogen and phosphorus concentrations were highly correlated (all Pearson's r > 0.80, S2 Table). Hence to avoid strong influences from correlated traits when assessing local community trait dispersion patterns, we selected WD, LA, SLA, LVD, dbh-H slope and C, for further analyses.

Bloomberg’s K for all measured PFT was <1 (Table 2), suggesting the evolutionary convergence of traits. However, LA and LVD were the only traits that had K values significantly different from null expectations.

### Trait community structure dispersion

**Fig 2:**
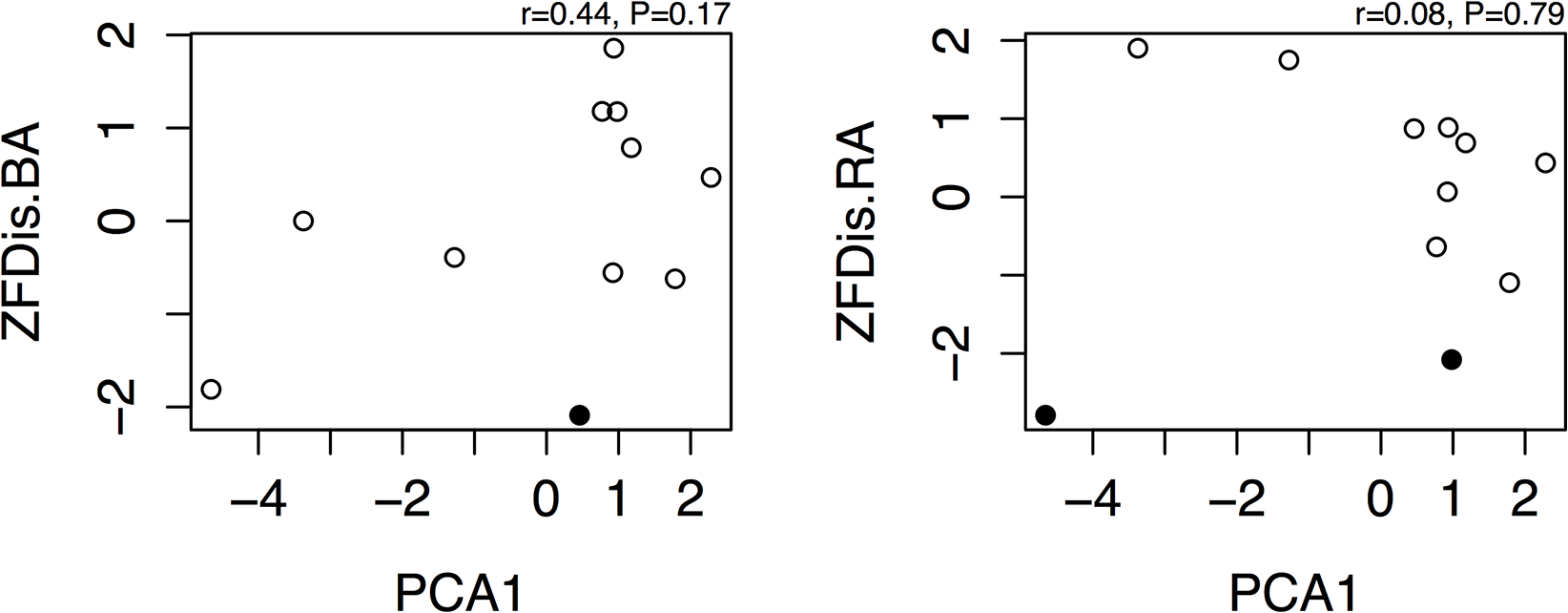
Correlation between the multivariate trait dispersion patterns (ZFDis) and soil parameters (PCA axis 1) for 11 plots at PKK. There was no significant correlation between either of the abundance-weight or basal area-weighted measures of trait dispersion patterns and the first soil PCA axis. Filled circles show the significant values of ZFDis (± 1.96; P<0.05) at the plot scale. PCA1 explained 59% of the variance in soil parameters. Multivariate ZFDis values were also not significantly correlated with PCA2 (not shown).

Out of the 11 plots, ZFDis values weighted by relative abundance (ZFDis.RA) were >1.96 (indicating significant clustering) only in two plots for LVD and one plot for SLA, LA, dbh-H and WD, respectively. Conversely, ZFDis.RA values were < -1.96 (indicating significant over-dispersion) in two plots for SLA and dbh-H, and one plot for LVD, C and WD, respectively. Least squares regression found that the ZFDis.RA values for all variables were not significantly associated with either PCA1 or PCA2 (S3 and S4 Figs) ZFDis values weighted by basal area (ZFDis.BA) were >1.96 in two plots for SLA and in one plot for WD and C, respectively, and <-1.96 in one plot for LA, C and WD. Least squares regression found that the ZFDis.BA values for all variables were not significantly associated with either PCA1 or PCA2, except for a significant positive relationship between leaf C and PCA2 (S5 and S6 Figs). However, caution is warranted in drawing any conclusions from this relationship, because of the multiple tests employed. We also consider ZFDis of traits combined in a multivariate analysis. The multivariate ZFDis.RA and ZFDis.BA values of across plots were both positive and negative. However, only two plots of ZFDis.RA and one plot of ZFDis.BA were <-1.96, indicating significant over-dispersal. Least squares regression found that the multivariate ZFDis.RA and ZFDis.BA values were not significantly associated with either PCA1 or PCA2 (Fig 2).

At the smaller scale (10 x 10 m), out of 275 plots multivariate ZFDis values weighted by basal area were significantly clustered (>1.96) for 31 plots and significantly over-dispersed (< -1.96) for 29 plots. For individual traits, C was >1.96 for 35 plots and <-1.96 for 30 plots. LA was >1.96 for 4 plots and also <-1.96 for 4 plots. LVD >1.96 for 24 plots and <-1.96 for 22 plots. SLA was >1.96 for 15 plots and <-1.96 for 13 plots. WD was >1.96 for 12 plots and <-1.96 for 14 plots. The dbh-H slope did not show any significant values. Multivariate ZFDis values weighted by relative abundance were >1.96 for 26 plots and <-1.96 for 31 plots, respectively. For individual traits, relative abundance weighted ZFDis values of C were >1.96 for 35 plots and <-1.96 for 41 plots. LA was >1.96 for 4 plots and <-1.96 for 5 plots. ZFDis values of LVD were >1.96 for 24 plots and <-1.96 for 18 plots. SLA was >1.96 for 13 plots and <-1.96 for 14 plots. WD was >1.96 for 27 plots and <-1.96 for 24 plots. The dbh-H slope was again not significantly distributed in any plot. Thus, there was no consistent pattern of trait dispersion for any individual trait or for the multivariate analyses at either the plot or subplot scales.

### Phylogenetic community structure dispersion

**Fig 3:**
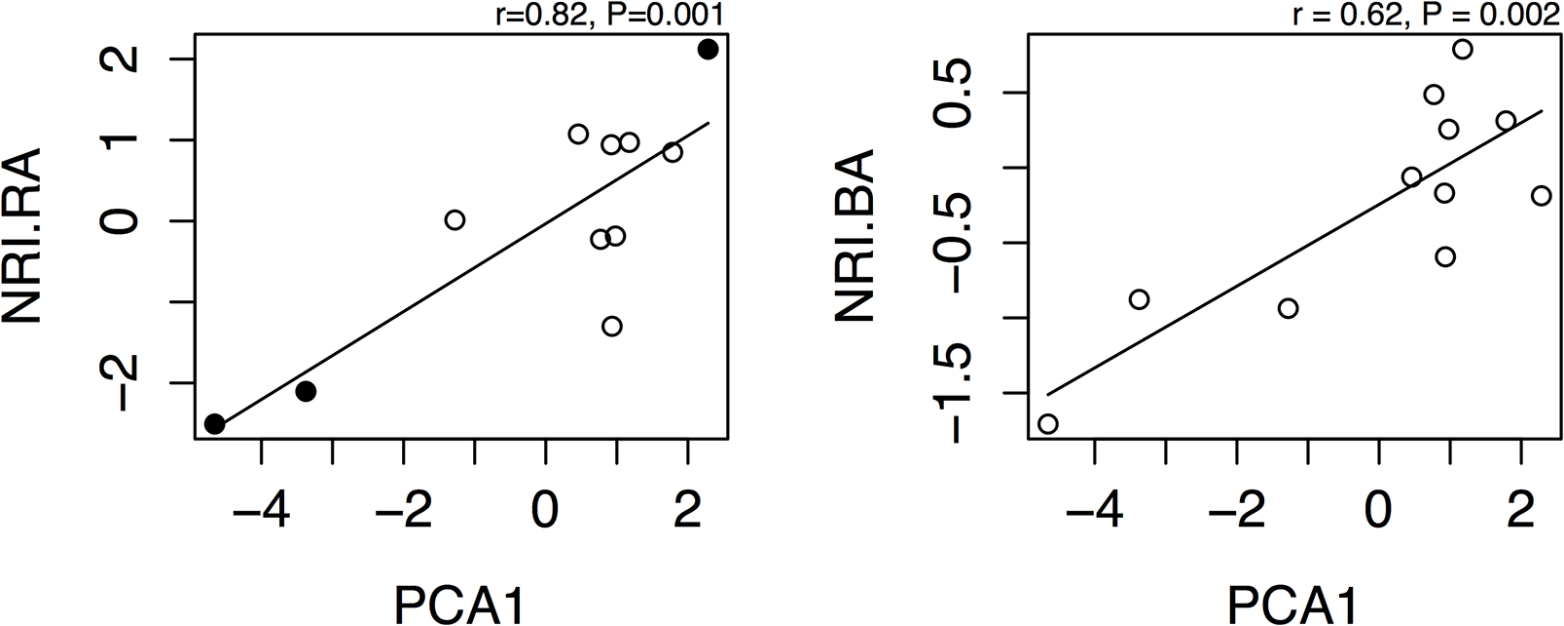
Correlation between local community phylogenetic structure and soil parameters (PCA axis 1) for 11 plots at PKK. Both relative abundance and basal area weighted values were significantly correlated with the first soil PCA axis (r = 0.82, P = 0.001 and r = 0.62, P = 0.002). There was no significant correlation between either of the phylogenetic dispersion patterns and PCA2 (not shown). Filled circles show significant values of NRI at the plot level (± 1.96; P<0.05). PCA1 explained 59% of the variance in soil parameters.

Across the 11 plots, community phylogenetic structure weighted by relative abundance (NRI.RA) was clustered in four plots and over-dispersed in seven plots. However, in only one plot was it significantly clustered and in only two plots was it significantly over-dispersed (NRI ± 1.96). NRI weighted by basal area (NRI.BA) was clustered in six plots and over-dispersed in five plots but none of NRI.BA values were significantly different from zero. However, phylogenetic structure for both basal area and relative abundance weightings was significantly correlated with soil PCA1 (r > 0.8, P = 0.001, r > 0.62, P = 0.002, respectively) (Fig 3), but not PCA2.

At the 10 x 10 m subplot scale, NRI.RA was significantly clustered in 8 out of 275 plots. NRI.BA was significantly clustered in 10 plots. None of NRI.RA and NRI.BA values were significantly over-dispersed.

### Functional similarity, phylogenetic similarity and soil gradients

Mantel tests indicated that functional trait similarity across plots was strongly correlated with similarity in soil conditions (r = 0.23, p = 0.03). However, there was no significant correlation between phylogenetic similarity and soil gradients (r = -0.21, p = 0.83).

## Discussion

Our correspondence analysis suggested that community composition at PKK was strongly determined by variation in soil parameters. However, we found no evidence for clustering of traits across local communities in either the individual trait analyses or in the multivariate analyses. Different traits in different plots varied from being significantly clustered to significantly over-dispersed at both plot (50 x 50 m) and subplot (10 x 10 m) scales. Meanwhile at the landscape level, there was no significant pattern of trait dispersion associated with variation in soil parameters. For example, there was no tendency for plots on sandier soils to be more functionally clustered, as we hypothesized. At PKK there is strong variation in soil conditions, which is believed to underlie the distinct forest formations observed, and our CCA results appear to confirm this. In addition, Mantel tests revealed that our matrices of functional trait distances and environmental distance (soil parameters) were significantly correlated, suggesting that variation in soil parameters and functional traits might be related. One possibility is that among adult trees functional traits may not be as well differentiated as among seedlings and saplings, which are the stages most relevant to habitat selection in plants. However, previous studies have shown that, although there is ontogenic change in functional traits, there is good correlation between juvenile and adult traits [36]. Another possibility is that plant community assembly across the soil gradient is being driven by some plant functional trait, or suite of traits, that we did not measure. Although we included key traits from both the leaf economic spectrum and the wood economic spectrum, the deep phylogenetic coverage of our community might have meant that our focus was on traits that were comparatively recently evolved and less important in community assembly in this case. Most of the traits in our analysis had a weakly convergent phylogenetic signal (K ranged from 0.034–0.054). Results elsewhere have suggested that a weak phylogenetic signal may cause a lack of concordance between trait dispersion and phylogenetic dispersion patterns [37, 38, 34]. For example, Swenson & Enquist [37] suggested that the lack of phylogenetic signal among traits on the phylogeny tree could lead to an inconsistent phylogenetic dispersion pattern. They also suggested that the results might be made more consistent if one examines the phylogenetic signal of traits at different phylogenetic depths, but we were not able to include this analysis in our study because of the limited number of species.

Across plots there was a mixture of phylogenetic clustering and over-dispersal. However, there was a strong correlation between the community phylogenetic structure and soil PCA1 and the most phylogenetically clustered plots were those with sandier soils and lower pH. These were also the same plots that were predicted to have higher levels of environmental filtering. These results tend to support the conjecture that community assemble in PKK may reflect a deeper phylogenetic structure. The greater community phylogenetic clustering on sandier soils may indicate the importance of more conserved traits in plant community assemble, while the influence of selection at the tips of the phylogeny is driving the convergent phylogenetic signal of the traits we measured.

Environmental filtering tends to increase functional similarity of the species within communities and lead to trait convergence, while competitive exclusion tends to limit functional similarity of the species within communities and lead to trait divergence [8, 39]. Carvender-Bares et al [8] found that convergent traits were strongly linked to environmental filtering and phenotypically similar species showed higher co-occurrence than expected by chance. We found evidence that the composition of trees in local communities at PKK was determined by soil conditions. However, our functional trait analyses did not support the hypothesis that environmental filtering was a dominant ecological process at our site. Nevertheless, we found evidence that communities on sandier soils were more phylogenetically clustered. Moreover, Mantel tests on distance matrices revealed a significant correlation between trait similarity and soil similarity across plots. This suggests that plant associations at PKK may be determined by deeper phylogenetically conserved traits that we did not measure, and that the functional traits we focused on may be responding to finer scale ecological processes within the forest formations at PKK. To examine this hypothesis would necessitate a much greater sampling effort than was possible in our study. Our study illustrates some of the pitfalls of combining trait and phylogenetic approaches to study plant community assembly. Specifically, non-significant or otherwise equivocal findings may be open to various interpretations, because of the complexities of integrating two multi-scale processes.

## Supporting Information

S1 Fig Principle component analysis of soil parameters. PCA1 accounted for 59% of the variance in measured soil parameters. Sand and pH were positively associated with PCA1, whereas P, K2O and soil silt and clay content were negatively associated with PCA1. PCA2 accounted for a further 25% of the variance in measured soil parameters. OM and N concentration were strongly negatively associated with PCA2.
(TIFF)

S2 Fig The phylogenetic tree used in the analyses of phylogenetic dispersion for the tree community at PKK. It was constructed using rbcL and matK genes downloaded from GeneBank for 115 species out of 123 species that occurred in the community.
(TIFF)

S3 Fig Variation of individual trait dispersion patterns weighted by relative abundance (ZFDis.RA) against soil parameters (PCA axes 1). The dispersion patterns of individual traits were correlated with PCA1 except WD.
(TIFF)

S4 Fig The relationship of ZFDis.RA of individual traits with PCA2. The dispersion patterns of individual traits weighted by relative abundance also correlated with PCA2. Again ZFDis.RA of WD did not change along soil parameters.
(TIFF)

S5 Fig Variation of individual dispersion patterns weighted by basal area (ZFDis.BA) across soil PCA axis 1. The ZFDis.BA of C, SLA and LVD were correlated with PCA1 but not LA, dbh-H and WD.
(TIFF)

S6 Fig The relationship of ZFDis.BA of each trait across soil PCA axis 2. The ZFDis.BA of WD and LVD were not correlated with PCA2.
(TIFF)

S1 Table List of tree species included in this study and the corresponding accession number for the DNA sequences downloaded from GenBank. (DOC)

S2 Table Pairwise correlations among plant functional traits at PKK. Significant correlation values (P < 0.05) are indicated in bold. Trait abbreviations as in Table 2.
(DOC)
