# Supplementary figures and images for: Using Plant Functional Traits and Phylogenies to Understand Patterns of Plant Community Assembly in a Seasonal Tropical Forest in Lao PDR
Source: PLoS One. 2015 Jun 26;10(6):e0130151. doi: 10.1371/journal.pone.0130151 (PMC4482738; doi:10.1371/journal.pone.0130151)

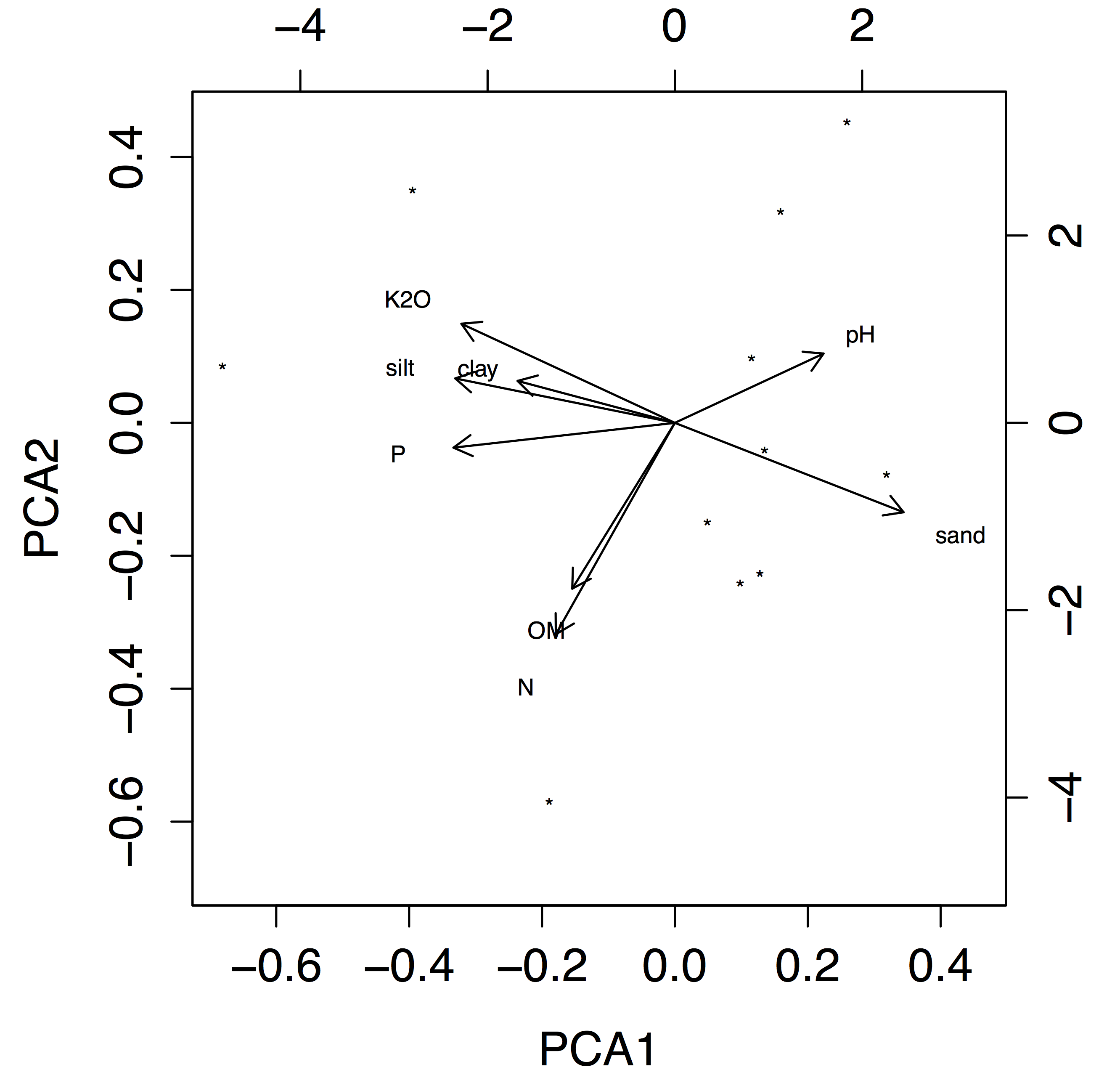

Supplement: S1 Fig — PCA1 accounted for 59% of the variance in measured soil parameters. Sand and pH were positively associated with PCA1, whereas P, K2O and soil silt and clay content were negatively associated with PCA1. PCA2 accounted for a further 25% of the variance in measured soil parameters. OM and N concentration were strongly negatively associated with PCA2. (TIFF) [file pone.0130151.s001.tiff]

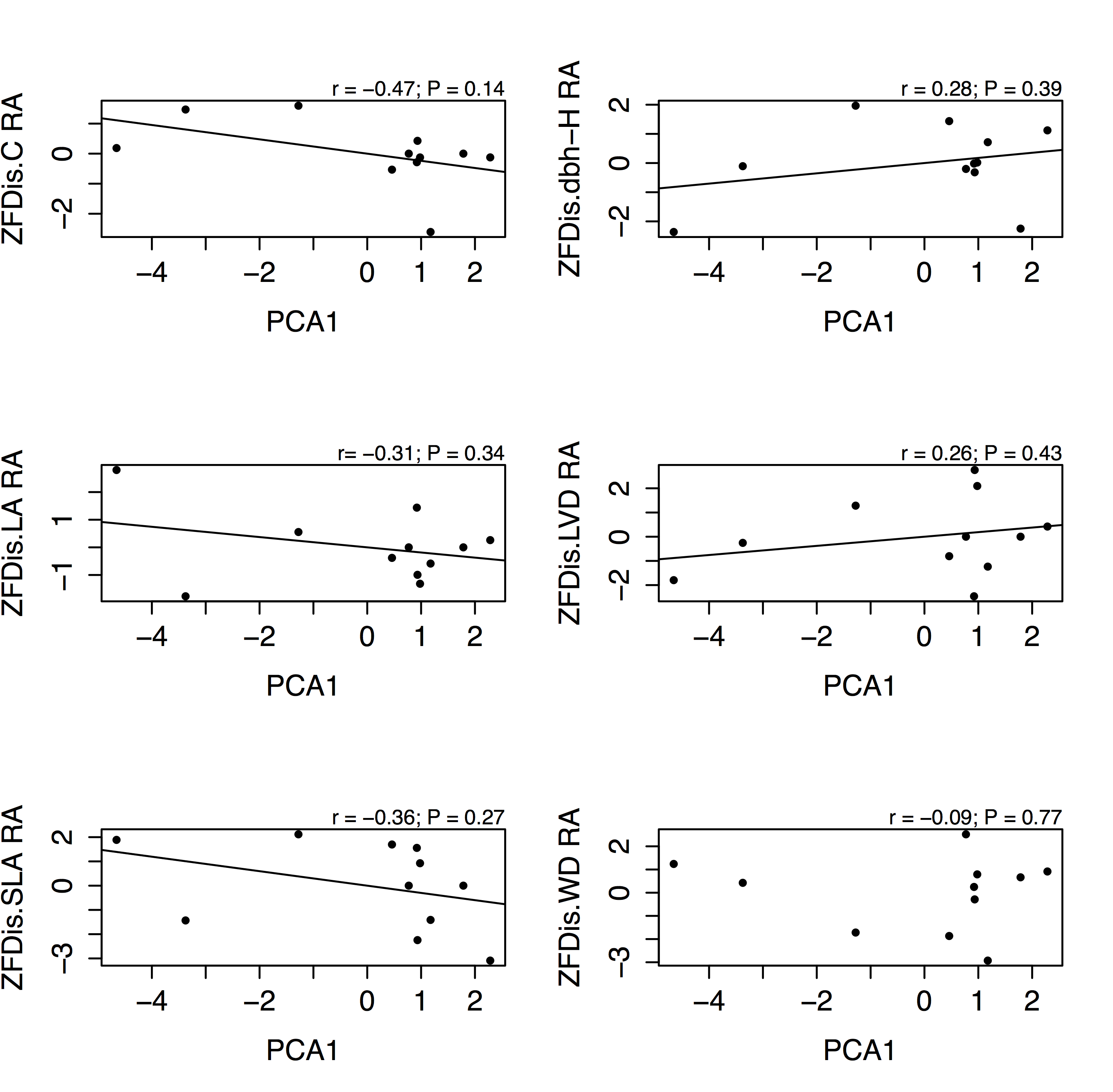

Supplement: S3 Fig — The dispersion patterns of individual traits were correlated with PCA1 except WD. (TIFF) [file pone.0130151.s003.tiff]

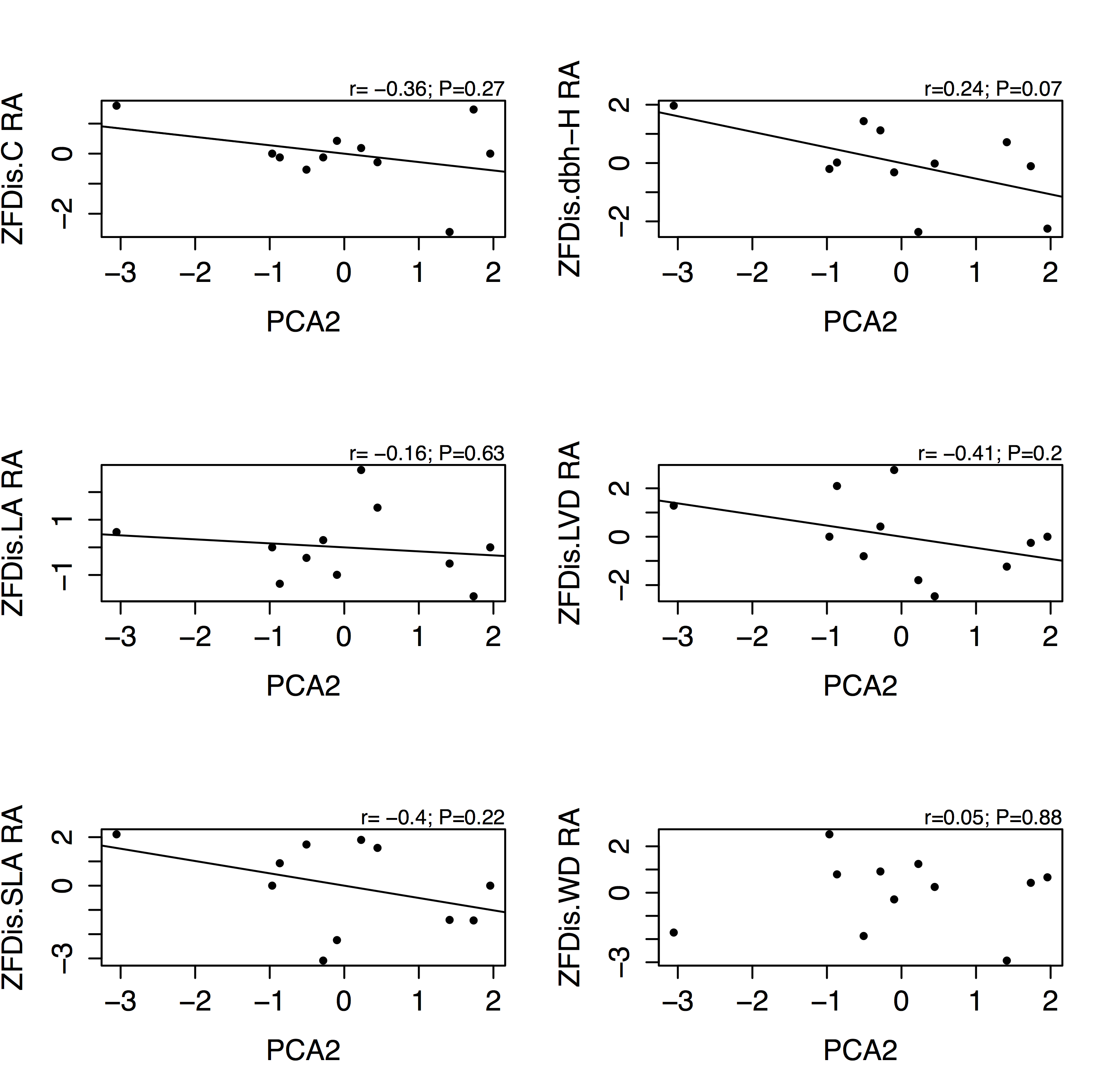

Supplement: S4 Fig — The dispersion patterns of individual traits weighted by relative abundance also correlated with PCA2. Again ZFDis.RA of WD did not change along soil parameters. (TIFF) [file pone.0130151.s004.tiff]

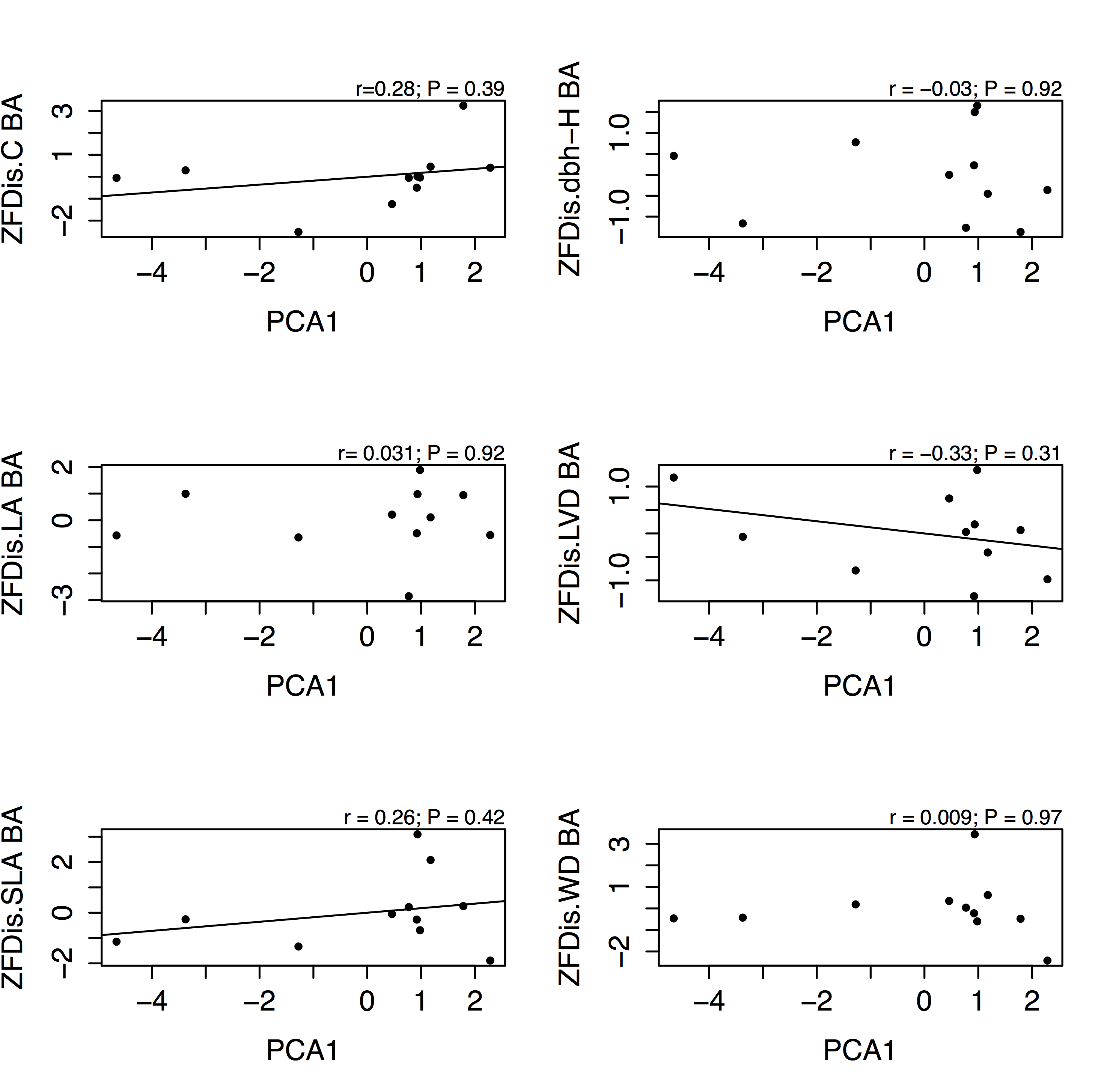

Supplement: S5 Fig — The ZFDis.BA of C, SLA and LVD were correlated with PCA1 but not LA, dbh-H and WD. (TIFF) [file pone.0130151.s005.tiff]

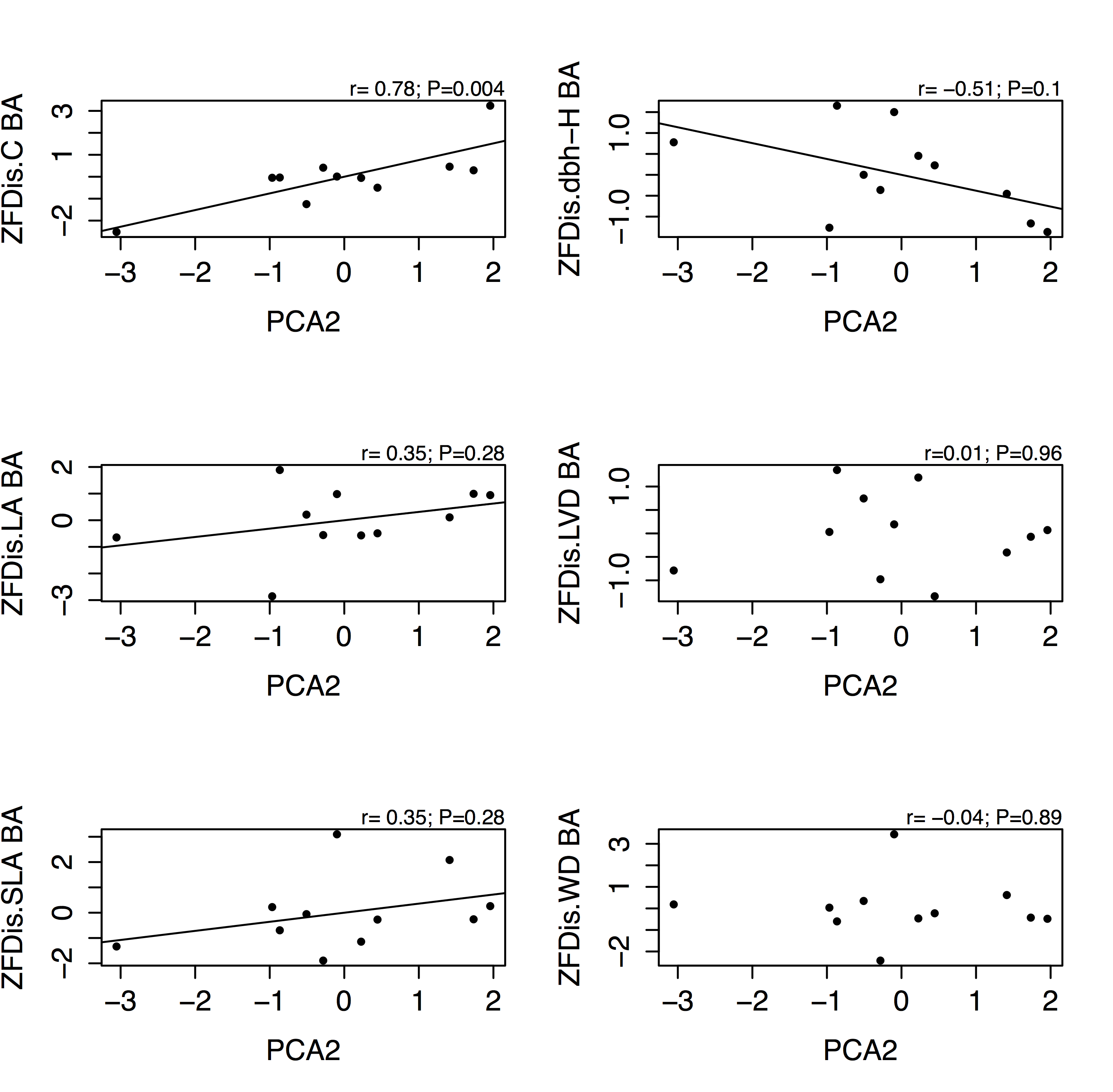

Supplement: S6 Fig — The ZFDis.BA of WD and LVD were not correlated with PCA2. (TIFF) [file pone.0130151.s006.tiff]
