# Supplementary material for: Using Plant Functional Traits and Phylogenies to Understand Patterns of Plant Community Assembly in a Seasonal Tropical Forest in Lao PDR
Source: PLoS One. 2015 Jun 26;10(6):e0130151. doi: 10.1371/journal.pone.0130151 (PMC4482738; doi:10.1371/journal.pone.0130151)
Supplement: S1 Table — (DOC) [file pone.0130151.s007.doc]

**S1 Table.** List of tree species included in this study and the corresponding accession number for the DNA sequences downloaded from GenBank.

| **Species** | **Family** | **Accession number for matK** |
| --- | --- | --- |
| *Gonocaryum lobbianum* | Cardiopteridaceae | GQ983654.1 |
| *Euonymus sp* | Celastraceae | HQ393842.1 |
| *Euonymus sp 1* | Celastraceae | HQ393836.1 |
| *Diospyros mollis* | Ebenaceae | AB174996.1 |
| *Diospyros pendula* | Ebenaceae | GU471729.1 |
| *Diospyros sp2* | Ebenaceae | GU471724.1 |
| *Diospyros sp4* | Ebenaceae | GU471722.1 |
| *Diospyros sp5* | Ebenaceae | GU471725.1 |
| *Barringtonia macrostachya* | Barringtoniaceae | JN183985.1 |
| *Styrax sp* | Styracaceae | HQ427282.1 |
| *Symplocos sp* | Symplocaceae | AF380108.1 |
| *Schima wallichii* | Theaceae | AF380100.1 |
| *Adinandra sp* | Theaceae | AF380069.1 |
| *Sindora laotica* | Fabaceae | EU362048.1 |
| *Sindora siamensis* | Fabaceae | AB924718.1 |
| *Ormosia cambodiana* | Fabaceae | JX295945.1 |
| *Ormosia sp1* | Fabaceae | JX295944.1 |
| *Peltophorum dasyrrhachis* | Fabaceae | AB924841.1 |
| *Dalbergia oliveri* | Fabaceae | HG326218.1 |
| *Dialium indum* | Fabaceae | AB924737.1 |
| *Xylia xylocarpa* | Fabaceae | AB924808.1 |
| *Aidia sp* | Rubiaceae | HQ427347.1 |
| *Canthium glabrum* | Rubiaceae | HQ415390.1 |
| *Canthium sp1* | Rubiaceae | AB925271.1 |
| *Canthium sp2* | Rubiaceae | JX518137.1 |
| *Gardenia annamensis* | Rubiaceae | KC699247.1 |
| *Gardenia sp1* | Rubiaceae | KC699249.1 |
| *Gardenia sp2* | Rubiaceae | KC699241.1 |
| *Gardenia sp3* | Rubiaceae | AB924827.1 |
| *Gardenia sp4* | Rubiaceae | AB924763.1 |
| *Hunteria zeylanica* | Apocynaceae | JX517717.1 |
| *Tarenna asiatica* | Rubiaceae | AB925104.1 |
| *Rothmannia attopevensis* | Rubiaceae | JX518115.1 |
| *Vitex pinnata* | Lamiaceae | AB925225.1 |
| *Chionanthus ramiflorus* | Oleaceae | AB924980.1 |
| *Cryptocarya amygdalina* | Lauraceae | AM889701.1 |
| *Cryptocarya sp* | Lauraceae | AM889702.1 |
| *Cinnamomum bejolghota* | Lauraceae | AB925175.1 |
| *Cinnamomum iners* | Lauraceae | JQ435501.1 |
| *Dehaasia sp* | Lauraceae | AB924704.1 |
| *Alphonsea gaudichaudiana* | Annonaceae | AY518811.1 |
| *Alphonsea sp* | Annonaceae | AY518810.1 |
| *Alphonsea sp 1* | Annonaceae | AY518809.1 |
| *Knema sp* | Myristicaceae | AB924868.1 |
| *Horsfieldia amygdalina* | Myristicaceae | GQ248135.1 |
| *Hydnocarpus ilicifolia* | Achariaceae | EF135551.1 |
| *Garcinia sp1* | Clusiaceae | JN564048.1 |
| *Garcinia sp2* | Clusiaceae | JN564049.1 |
| *Garcinia multiflora* | Clusiaceae | HQ331610.1 |
| *Garcinia xanthochymus* | Clusiaceae | AB924823.1 |
| *Aporosa sp1* | Phyllanthaceae | AB924795.1 |
| *Chaetocarpus castanocarpus* | Euphorbiaceae | AB924729.1 |
| *Croton argyratus* | Euphorbiaceae | KC627680.1 |
| *Croton kongensis* | Euphorbiaceae | GQ434077.1 |
| *Triadica sp1* | Euphorbiaceae | HQ415366.1 |
| *Cratoxylum cochinchinense* | Hypericaceae | AB233787.1 |
| *Cratoxylum formosum* | Hypericaceae | AB924785.1 |
| *Irvingia malayana* | Irvingiaceae | AB233788.1 |
| *Microdesmis caseariifolia* | Pandaceae | AB925019.1 |
| *Rinorea boissieui* | Violaceae | JQ626350.1 |
| *Hopea ferrea* | Dipterocarpaceae | AB006384.1 |
| *Hopea sp* | Dipterocarpaceae | AB246461.1 |
| *Hopea odorata* | Dipterocarpaceae | AB006385.1 |
| *Dipterocarpus costatus* | Dipterocarpaceae | KJ611241.1 |
| *Shorea thorelii* | Dipterocarpaceae | KJ611242.1 |
| *Vatica harmandiana* | Dipterocarpaceae | AB246420.1 |
| *Vatica odorata* | Dipterocarpaceae | AB924681.1 |
| *Elaeocarpus sp* | Elaeocarpaceae | AB936009.1 |
| *Elaeocarpus tectorius* | Elaeocarpaceae | AB936047.1 |
| *Microcos tomentosa* | Malvaceae | AB924802.1 |
| *Microcos paniculata* | Malvaceae | JN407216.1 |
| *Bombax anceps* | Malvaceae | AB924835.1 |
| *Pterospermum sp1* | Sterculiaceae | HQ415246.1 |
| *Sterculia sp2* | Sterculiaceae | JX518037.1 |
| *Aquilaria sp* | Thymeleaceae | HQ415244.1 |
| *Lagerstroemia balansae* | Lythraceae | AB924736.1 |
| *Lagerstroemia calyculata* | Lythraceae | AB925059.1 |
| *Lagerstroemia sp2* | Lythraceae | AB924955.1 |
| *Memecylon ovatum* | Melastomataceae | AB924697.1 |
| *Memecylon sp3* | Melastomataceae | KF895404.1 |
| *Memecylon sp4* | Melastomataceae | KF895408.1 |
| *Syzygium cinereum* | Myrtaceae | JX495762.1 |
| *Syzygium lineatum* | Myrtaceae | JQ588516.1 |
| *Syzygium sp* | Myrtaceae | JF270959.1 |
| *Syzygium sp1* | Myrtaceae | JN564161.1 |
| *Syzygium sp2* | Myrtaceae | HQ415318.1 |
| *Syzygium sp3* | Myrtaceae | GU135056.1 |
| *Syzygium syzygioides* | Myrtaceae | AB924734.1 |
| *Syzygium grande* | Myrtaceae | AB925279.1 |
| *Xanthophyllum sp1* | Polygalaceae | HQ415290.1 |
| *Xanthophyllum sp2* | Polygalaceae | AB924705.1 |
| *Xanthophyllum virens* | Polygalaceae | JN564166.1 |
| *Xanthophyllum flavescens* | Polygalaceae | AB924997.1 |
| ***Species*** | **Family** | **Accession number for rbcL** |
| *Parinari anamensis* | Chrysobalanaceae | AB925417.1 |
| *Artocarpus sp1* | Moraceae | KJ767825.1 |
| *Artocarpus lakoocha* | Moraceae | JF738388.1 |
| *Streblus ilicifolius* | Moraceae | AF500353.1 |
| *Streblus sp* | Moraceae | GQ436643.1 |
| *Streblus taxoides* | Moraceae | GQ436641.1 |
| *Gironniera nervosa* | Cannabaceae | JN040336.1 |
| *Prunus sp* | Rosaceae | KF154867.1 |
| *Canarium sp* | Burseraceae | GU246029.1 |
| *Canarium sp 1* | Burseraceae | FJ466642.1 |
| *Walsura sp* | Meliaceae | AY128246.1 |
| *Walsura trichostemon* | Meliaceae | AB925350.1 |
| *Xerospermum noronhianum* | Sapindaceae | AB925339.1 |
| *Mangifera odorata* | Anacardiaceae | GQ436547.1 |
| *Buchanania sp* | Anacardiaceae | GU935418.1 |
| *Aglaia sp1* | Meliaceae | FN599442.1 |
| *Aglaia grandis* | Meliaceae | AY128211.1 |
| *Aphanamixis polystachya* | Meliaceae | AY128213.1 |
| *Sandoricum koetjape* | Meliaceae | AB925638.1 |
| *Lepisanthes sp* | Sapindaceae | AY724360.1 |
| *Eurycoma longifolia* | Simaroubaceae | EU042996.1 |
| *Glycosmis cochinchinensis* | Rutaceae | JX144155.1 |
