# Supplementary material for: Using Plant Functional Traits and Phylogenies to Understand Patterns of Plant Community Assembly in a Seasonal Tropical Forest in Lao PDR
Source: PLoS One. 2015 Jun 26;10(6):e0130151. doi: 10.1371/journal.pone.0130151 (PMC4482738; doi:10.1371/journal.pone.0130151)
Supplement: S2 Table — Significant correlation values (P < 0.05) are indicated in bold. Trait abbreviations as in Table 2. (DOC) [file pone.0130151.s008.doc]

**S2 Table. Pairwise correlations among plant functional traits at PKK.**

|  | WD | LA | SLA | LT | LVD | DBH | H | CW | BA | C | N |
| --- | --- | --- | --- | --- | --- | --- | --- | --- | --- | --- | --- |
| LA | -0.29*** | 1.00 |  |  |  |  |  |  |  |  |  |
| SLA | -0.22** | 0.14 | 1.00 |  |  |  |  |  |  |  |  |
| LT | 0.14 | -0.003 | -0.39**** | 1.00 |  |  |  |  |  |  |  |
| LVD | 0.01 | 0.22** | -0.06 | -0.25** | 1.00 |  |  |  |  |  |  |
| DBH | 0.15 | -0.002 | -0.02 | 0.18* | 0.22** | 1.00 |  |  |  |  |  |
| H | 0.08 | -0.003 | 0.07 | -0.03 | 0.28*** | 0.69**** | 1.00 |  |  |  |  |
| CW | 0.13 | 0.07 | 0.05 | -0.01 | 0.22** | 0.80**** | 0.73**** | 1.00 |  |  |  |
| BA | 0.21 | -0.03 | -0.02 | 0.17 | 0.22** | 0.96**** | 0.63**** | 0.75**** | 1.00 |  |  |
| C | 0.10 | -0.20 | -0.16 | 0.14 | 0.02 | -0.06 | -0.13 | -0.20* | -0.03 | 1.00 |  |
| N | -0.01 | -0.14 | 0.09 | -0.01 | -0.05 | -0.09 | -0.10 | -0.19* | -0.06 | 0.86**** | 1.00 |
| P | -0.02 | -0.14 | 0.08 | -0.03 | -0.02 | -0.15 | -0.18 | -0.23** | -0.12 | 0.80**** | 0.90**** |

**Significant correlation values (P < 0.05) are indicated in bold. Trait abbreviations as in Table 2.**

** P < 0.05; ** P < 0.01; *** P < 0.001; **** P < 0.0001*
